# Protein tyrosine phosphatase-1B regulates the tyrosine phosphorylation of the adapter Grb2-associated binder 1 (Gab1) in the retina

**DOI:** 10.1186/1478-811X-11-20

**Published:** 2013-03-22

**Authors:** Ammaji Rajala, Ashok K Dilly, Raju VS Rajala

**Affiliations:** 1 Departments of Ophthalmology, University of Oklahoma Health Sciences Center, Oklahoma City, OK, 73104, USA; 2 Departments of Physiology, University of Oklahoma Health Sciences Center, Oklahoma City, OK, 73104, USA; 3 Departments of Cell Biology, University of Oklahoma Health Sciences Center, Oklahoma City, OK, 73104, USA; 4 Dean A. McGee Eye Institute, University of Oklahoma Health Sciences Center, 608 Stanton L. Young Blvd., Oklahoma City, OK, 73104, USA; 5 Present address: Wayne State University School of Medicine, Detroit, MI, 48202, USA

**Keywords:** Adapter protein, Gab1, PTP1B, Phosphorylation, Retina, Photoreceptors

## Abstract

**Background:**

Gab1 (Grb2-associated binder 1) is a key coordinator that belongs to the insulin receptor substrate-1 like family of adaptor molecules and is tyrosine phosphorylated in response to various growth factors, cytokines, and numerous other molecules. Tyrosine phosphorylated Gab1 is able to recruit a number of signaling effectors including PI3K, SHP2 and PLC-γ. In this study, we characterized the localization and regulation of tyrosine phosphorylation of Gab1 in the retina.

**Results:**

Our immuno localization studies suggest that Gab1 is expressed in rod photoreceptor inner segments. We found that hydrogen peroxide activates the tyrosine phosphorylation of Gab1 *ex vivo* and hydrogen peroxide has been shown to inhibit the protein tyrosine phosphatase PTP1B activity. We found a stable association between the D181A substrate trap mutant of PTP1B and Gab1. Our studies suggest that PTP1B interacts with Gab1 through Tyrosine 83 and this residue may be the major PTP1B target residue on Gab1. We also found that Gab1 undergoes a light-dependent tyrosine phosphorylation and PTP1B regulates the phosphorylation state of Gab1. Consistent with these observations, we found an enhanced Gab1 tyrosine phosphorylation in PTP1B deficient mice and also in retinas treated *ex vivo* with a PTP1B specific allosteric inhibitor.

**Conclusions:**

Our laboratory has previously reported that retinas deficient of PTP1B are resistant to light damage compared to wild type mice. Since Gab1 is negatively regulated by PTP1B, a part of the retinal neuroprotective effect we have observed previously in PTP1B deficient mice could be contributed by Gab1 as well. In summary, our data suggest that PTP1B regulates the phosphorylation state of retinal Gab1 *in vivo.*

## Background

Gab1 (Grb2-assoicated binder 1) is a member of a small group of scaffolding adapters that includes *Drosophila melanogaster* Dos (Daughter of Sevenless), the *Caneorhabditis elegans* homolog Soc1 (Suppressor-Of Clear), and mammalian Gab2 and Gab3 [1-8]. These proteins contain an amino-terminal PH domain, several proline-rich sequences, and multiple binding sites for SH2-domain containing proteins. Upon stimulation of appropriate cells with any of a number of receptor tyrosine kinase ligands, including epidermal growth factor (EGF), hepatocyte growth factor (HGF), platelet-derived growth factor (PDGF), nerve growth factor (NGF), and insulin or insulin-like growth factor 1 (IGF-1), Gab1 rapidly becomes tyrosine phosphorylated [3,8-11]. Tyrosine phosphorylated Gab1 binds multiple signal-relay molecules, including the p85 subunit of phosphoinositide 3^′^-kinase, Shc, and the protein tyrosine phosphatase (PTP) Shp2 [3,8,12,13]. In addition to the binding sites for SHP2 and p85, both Gab1 and Gab2 contain numerous YxxP motifs, potential binding sites for the SH2 domain of PLCγ or Crk family proteins [14]. Further, Grb2 binds to Gab proteins via its C-terminal SH3 domain in a phospho-tyrosine independent manner [15,16].

The physical association between p85 and Gab1 or Gab2 is critical in mediating the PI3K/Akt signaling pathway induced by a variety of stimuli [9,10,17-22]. Overexpression of Gab potentiates FGF-induced Akt activity, whereas overexpression of the p85 binding mutant of Gab1 results in decreased Akt activation [21]. The same mutant is also unable to provide anti-apoptotic signal in response to nerve growth factor stimulation [9]. Mutation in the p85-binding sites of Gab2 was found to impair the ability of IL-3 to activate Akt and to induce cell growth [18]. These studies clearly suggest that Gab-p85 interaction plays an important role in activating the PI3K/Akt pathway in mammalian cells. The activation of PI3K leads to the production of PIP3, which in turn can bind to the PH domain of Gab proteins and presumably promote further activation of PI3K, a positive feedback loop which could be formed to amplify the signal through the Gab proteins [10]. The EGF-dependent positive feedback loop is negatively regulated by SHP2 by dephosphorylating Gab1-p85 binding sites, thereby terminating the Gab1-P3K positive loop [23].

Many retinal degenerative diseases show an early loss of rod cells followed by cone cell loss, and the pathological phenotype for this loss is apoptosis [24-26]. Blocking of photoreceptor apoptosis is one of the possible therapeutic approaches to protect the morphology and function of the retina and prolong the period of useful vision in patients. The mechanisms of protection are still largely unknown but may involve differential intercellular signaling cascade. We and others have shown that PI3K activation is neuroprotective [27,28]. Hepatocyte growth factor (HGF) is shown to protect light-induced photoreceptor degeneration [29] and retinal ischemia-reperfusion injury [30] and also attenuates the ceramide-induced apoptosis in retina [31]. All these studies clearly suggest that HGF possesses both neuroprotective and anti-oxidant properties [29,31]; however, the molecular mechanism behind the neuroprotective effect remains unclear. Both HGF and its receptor c-Met are expressed in the retina [32]. Interaction between Gab1 and the cMet receptor tyrosine kinase is responsible for epithelial morphogenesis [33]. Upon interaction with cMet, Gab1 becomes phosphorylated on several tyrosine residues which, in turn, recruit a number of signaling effectors, including PI3K, SHP2, and PLC-γ. Gab1 phosphorylation by cMet results in a sustained signal that mediates most of the downstream signaling pathways [34,35]. The association between protein tyrosine phosphatase-1B (PTP1B) and c-Met receptor in the modulation of corneal epithelial wound healing has been reported previously [36]. However, absolutely there are no data available on the expression and regulation of tyrosine phosphorylation of Gab1 in the retina. In this study we have examined the localization of Gab1 and how the phosphorylation state of Gab1 is regulated in the retina as the interaction of Gab1 with effector proteins is phosphorylation-dependent. Our studies suggest that Gab1 is predominantly localized to rod inner segments under both dark- and light-adapted conditions; however, the state of Gab1 phosphorylation is light-dependent. Our studies also suggest that protein tyrosine phosphatase, PTP1B, regulates the Gab1 phosphorylation *in vivo* as we found enhanced phosphorylation of Gab1 in PTP1B deficient mice and retinas treated *ex vivo* with a PTP1B specific inhibitor. We also found a region between 1–280 amino acids in Gab1 encompassing Y83 is required for PTP1B binding.

## Results

### Localization of Gab1 in the retina

**Figure 1:**
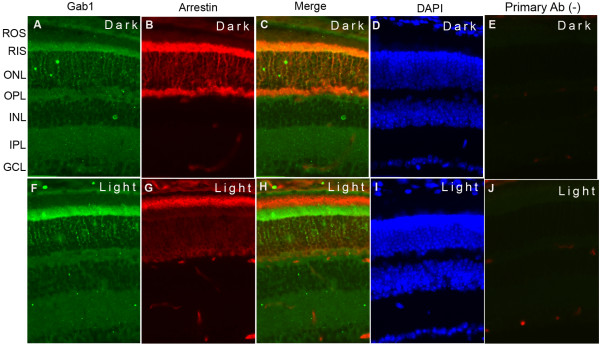
**Immunofluorescence analysis of Gab1 in rat retina**. Prefer-fixed sections of dark- (**A-E**) and light-adapted (**F-J**) rat retinas were stained for Gab1 (**A**, **F**), arrestin (**B**, **G**) and DAPI (**D**, **I**) and the immunofluorescence was analyzed by epifluorescence. Panel C and H represent the merge images of Gab1 and arrestin whereas panel **E** and **F** represent the omission of Gab1 antibody. ROS, rod outer segments; RIS, rod inner segments; ONL, outer nuclear layer; OPL, outer plexiform layer; INL, inner nuclear layer; IPL, inner plexiform layer; GCL, ganglion cell layer.

**Figure 2:**
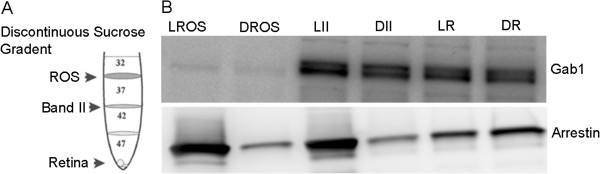
**Immunoblot analysis of Gab1 in various fractions of rat retina.** Rod outer segment membranes (ROS) were prepared from dark- and light-adapted rats on a discontinuous sucrose density gradient centrifugation (**A**). ROS (sucrose 32/37% interface), non-ROS membranes (sucrose 37/42% interface, band II) and rest of the retina (bottom of the gradient) were subjected to immunoblot analysis with anti-Gab1 and anti-arrestin (**B**) antibodies. LROS, light-adapted ROS; DROS, dark-adapted ROS; LII, light-adapted band II; DII, dark-adapted band II; LR, light-adapted rest of the retina; DR, dark-adapted rest of the retina;

Retinal sections from dark- and light-adapted (300 lux for 30 min) rats were subjected to immunohistochemistry with Gab1 and arrestin antibodies. Immunolocalization studies suggest that Gab1 is exclusively localized to rod inner segments (Figure 1A and F) and co-localizes with arrestin in dark-adapted retina (Figure 1C). The adaptability of animals to dark and light conditions is examined with arrestin immunolocalization. In dark-adapted retinas, arrestin is localized to the rod inner segments and the outer plexiform layer (Figure 1B), and upon light illumination arrestin is translocated to photoreceptor outer segments (Figure 1G). Our immunohistochemical data suggest that Gab1 predominantly localized to rod inner segments irrespective of dark or light adaptation (Figure 1A and F). Rod outer segments (DROS/LROS), band II (DII/LII) containing enriched inner segments and other retinal cells, and rest of the retina (DR/LR) fractions (Figure 2A) from dark- and light-adapted rats were subjected to immunoblot analysis with anti-Gab1 (Figure 2B) and anti-arrestin (Figure 2B) antibodies. In Figure 1 we show arrestin in light adapted retina in the ROS; however in Figure 2 arrestin was found in the LII fraction. In dark adapted retina arrestin was found in the RIS (Figure 1) but not in DII (Figure 2). This discrepancy is due to the affinity of arrestin towards photoactivated rhodopsin. It is a well know phenomenon that arrestin binds to photoactivated rhodopsin upon light illumination and in a dark-adapted retinas arrestin is soluble. We have employed a discontinuous sucrose density centrifugation which allows only obtaining membranes, hence we did not observe the presence of arrestin in DII. The results indicate that Gab1 is present in Band II and rest of the retina fractions and very low levels of Gab1 is present in ROS (Figure 2B). Collectively, these results suggest that Gab1 is predominantly expressed in rod inner segments.

### Light-dependent phosphorylation of Gab1

**Figure 3:**
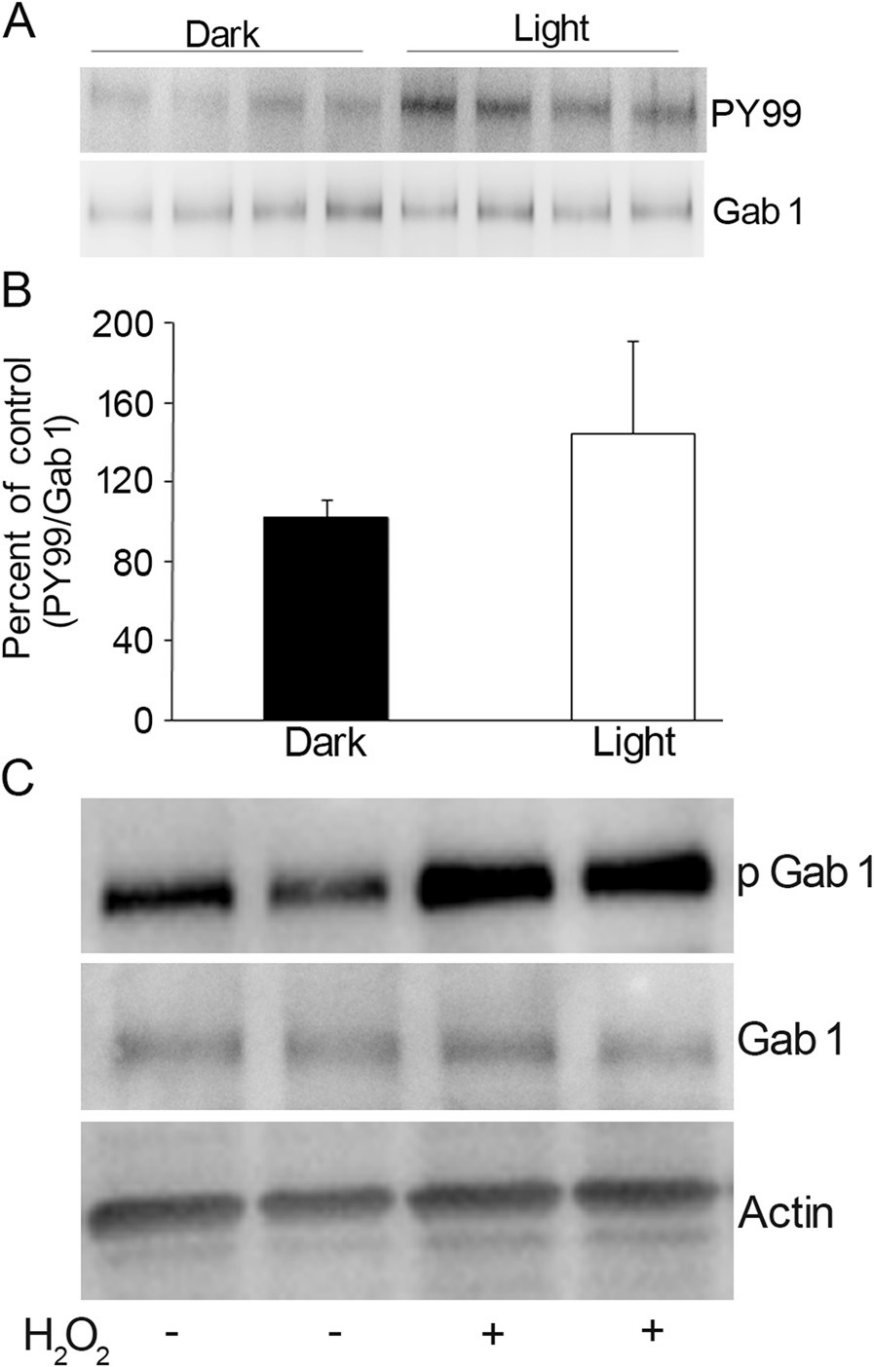
**Light-dependent phosphorylation of Gab1 in the retina.** Retina lysates from dark- and light-adapted rats were immunoprecipitated with Gab1 antibody followed by immunoblot analysis with anti-PY99 antibody (**A**). The blot was stripped and reprobed with anti-Gab1 antibody (**A**). Densitometric analysis of PY99 immunoblot was performed in the linear range of detection and absolute values were then normalized to Gab1 (**B**). Data are mean ± SD, n=4. H_2_O_2_-stimulated tyrosine phosphorylation of retinal Gab1. Retinal proteins from (two independent rats) controls and H_2_O_2 (_600 μM) treated *ex vivo* retinal explants were subjected to immuno blotting analysis with anti-pGab1, anti-Gab1 and anti-actin antibodies (**C**).

On the day of an experiment, rats were dark-adapted overnight and half were subjected to normal room light (~300 lux) for 30 min. Eyes were enucleated and the retinas were quickly removed and homogenized in homogenizing buffer [37]. The retina lysate was immunoprecipitated with anti-Gab1 antibody, followed by immunobot analysis with anti-PY99 and anti-Gab1 antibodies. The results indicate an increased level of Gab1 phosphorylation in light-adapted compared to dark-adapted retinas (Figure 3A). The blots were stripped and reprobed with total Gab1 to ensure equal amounts of Gab1 in both immunoprecipitates (Figure 3A). Densitometric analysis of PY99 immunoblot was performed in the linear range of detection and absolute values were then normalized to Gab1 (Figure 3B). These results suggest that phosphorylation of Gab1 is light-induced *in vivo.*

### Hydrogen peroxide activates the Gab1 phosphorylation

Previously H_2_O_2_ has been shown to induce the phosphorylation of Gab1 which results in the binding of SHP2 [38]. Therefore we have examined the Gab1 phosphorylation on Y627 (binding site of SHP2) residue in response to H_2_O_2_ in retinal *ex vivo* explants. To determine the effect of H_2_O_2_ on Gab1 phosphorylation, we incubated mouse retinal *ex vivo* explants for 10 min in the presence or absence of 600 μM H_2_O_2_. Retinal proteins were prepared and subjected to immunoblot analysis with anti-pGab1-Tyr^627^ antibody and the results indicate an increased phosphorylation of Gab1 was observed in H_2_O_2_ treated retinas compared to control retinas (Figure 3C) while the total Gab1 levels are unchanged (Figure 3C). The blot was reprobed with anti-actin antibody (Figure 3C) to ensure an equal amount of protein in each lane. These results suggest that H_2_O_2_ activates the Gab1 phosphorylation.

### Binding of Gab1 to p85 (N-SH2) domain of PI3K

**Figure 4:**
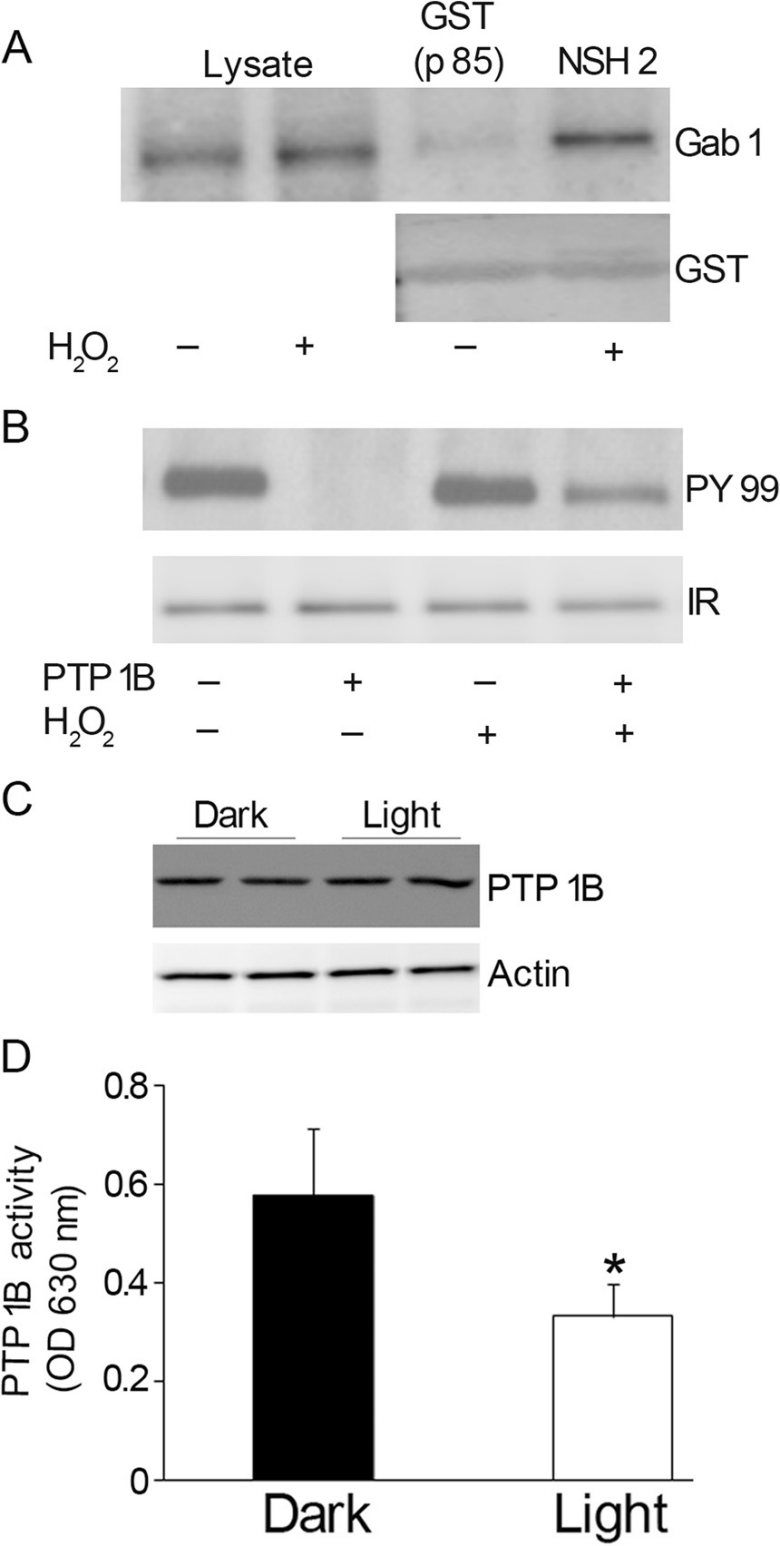
**Interaction of Gab1 with N-SH2 domain of p85 subunit of PI3K.** Retinal proteins from (two independent rats) controls and H_2_O_2_ (600 μM) treated *ex vivo* retinal explants were subjected to GST pull-down assay with N-SH2 domain of p85, followed by immunoblot analysis with anti-Gab1 antibody (**A**). The blot was reported with GST to ensure equal amount of fusion in each lane (A). PTP1B dephosphorylates the tyrosine phosphorylation of IR *in vitro***.** Rat retinas were dissected and incubated at 37°C for 5 min in DMEM medium in the presence or absence of insulin (1 μM). After incubation, the retinas were lysed and subjected to immunoprecipitation with anti-IRβ antibody. The anti-IRβ immune complexes were subjected to *in vitro* dephosphorylation by PTP1B in the presence or absence of H_2_O_2_ for 15 min at 37°C. The reaction after dephosphorylation was subjected to immunoblot analysis with anti-PY99 antibody (**B**). The blot was stripped and reprobed with anti-IRβ antibody to ensure equal amounts of protein in each immunoprecipitate (**B**). Inhibition of PTP1B activity in light-adapted retina. Retinas from each rat were immunoprecipitated with anti-PTP1B antibody and PTP1B activity measured (**D**). Data are mean ± SD, n=5, *p<0.05. Twenty μg of retinal proteins from dark- and light-adapted rat retinas (two independent rats) were immunoblotted with anti-PTP1B and anti-actin antibodies (**C**).

To further determine whether the activated Gab1 binds to p85 subunit of PI3K, we subjected the retinal lysates from control and H_2_O_2_-stimulated retinas to GST pull-down assay with GST N-SH2 domain of p85. The p85 N-SH2 domain of PI3K was able to pull down Gab1 from H_2_O_2_-treated retinas as detected on the immunoblot probed with anti-Gab1 antibody (Figure 4A). To ensure equal amounts of fusion proteins in each pull-down, Gab1 blot was reprobed with anti-GST antibody (Figure 4A). These results suggest that the p85 subunit of PI3K binds to Gab1 in H_2_O_2_-induced stress conditions. This experiment also suggests that in addition to the phosphorylation of SHP2 binding site on Gab1 (Y627), H_2_O_2_ also induces the phosphorylation of p85 subunit of PI3K binding sites on Gab1 (Y448; Y473 and Y590).

### Possible mechanism of H_2_O_2_-induced Gab1 activation

The exact mechanism of H_2_O_2_-induced Gab1 activation is not known. However, it has been shown previously that H_2_O_2_ inhibits the PTP1B activity [38,39]. We also tested in this study the H_2_O_2_-induced inhibition of PTP1B activity. We stimulated the rat retinas *ex vivo* with insulin, and the retinal lysates were immunoprecipitated with anti-IRβ antibody. The IR immunoprecipitates were subjected to dephosphorylation assay by PTP1B in the presence and absence of H_2_O_2_ followed by immunoblot analysis with anti-PY99 antibody. The results indicate that PTP1B dephosphorylates the IR and the dephosphorylation of IR by the PTP1B was partially prevented in the presence of H_2_O_2_ (Figure 4B). The observed activation of Gab1 in this study could be due to the inhibition of PTP1B activity and that Gab1 could be a substrate of PTP1B.

### Light-dependent inhibition of retinal PTP1B activity

To determine whether light regulates PTP1B activity, we immunoprecipitated PTP1B from lysates of dark- and light-adapted rat retinas and measured the PTP1B activity. The PTP1B activity was significantly greater in dark-adapted retinas than in the light-adapted retinas (Figure 4D). To determine whether this greater PTP1B activity was due to increased protein expression in the dark-adapted retinas, we subjected the proteins from dark- and light-adapted retinas to immunoblotting with anti-PTP1B antibody (Figure 4C). No significant differences in the expression of PTP1B was found between the dark- and light-adapted mouse retinas, suggesting that light regulates PTP1B activity *in vivo*.

### Identification of Gab1 as a substrate of PTP1B in vitro

**Figure 5:**
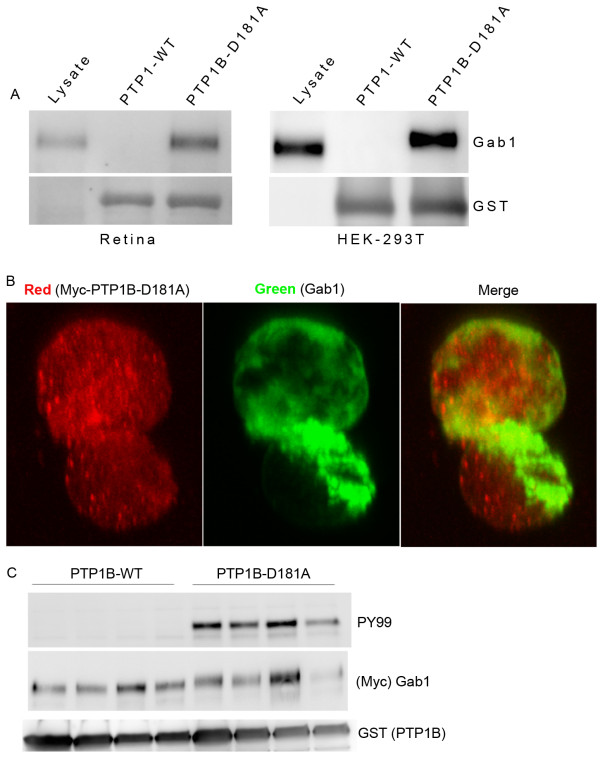
**Identification of Gab1 as PTP1B substrate with substrate trapping mutant technique.** Pervanadate-treated retinal *ex vivo* explants or Gab1 expressed HEK-293 T cell lysates were subjected to GST pull-down assay with either wild type PTP1B or mutant PTP1B-D181A followed by immuno blot analysis with anti-Gab1 antibody (**A**). The blot was reprobed with anti-GST antibody to ensure equal amounts of fusion in each pull-down (A). Lysate, retinal proteins and HEK-293 T cell expressed Gab1 were used as positive controls. Co-localization of D181A-PTP1B and Gab1. HEK-293 T cells cotransfected with Myc-tagged D181A-PTP1B and Gab1 expression plasmids were fixed with paraformaldehyde and processed for immunofluorescence and visualized by confocal microscopy. Cells were incubated with anti-Myc (red) and Gab1 (green) antibodies (**B**). Overexpressed PTP1B and Gab1 were visualized with fluorescein-conjugated sheep anti-mouse and Texas red-conjugated goat anti-rabbit antibodies, respectively. Right panel represents the merge image of D181A-PTP1B and Gab1. PTP1B dephosphorylates the Gab1 tyrosine phosphorylation *in vitro*. Myc-tagged Gab1 was transfected (four independent transfections) into HEK-293 T cells and the proteins were subjected to immunoprecipitation with anti-Myc antibody. The immune complexes were incubated with GST fusion proteins of either wild type PTP1B or catalytically inactive PTP1B (D181A) for 15 min at 30°C. At the end of the reaction, the immune complexes were subjected to SDS-PAGE followed by immunoblot analysis with anti-PY99 antibody (**C**). The blot was stripped and reprobed with anti-Myc and anti-GST antibodies (C).

Previously, Tonks group has discovered a mutation of the invariant catalytic acid (Asp-181 in PTP1B) that converts an extremely active enzyme into a “substrate-trap,” and with the advent of this mutant several PTP1B substrates have been identified [40,41]. To determine whether or not Gab1 is a substrate of PTP1B we transiently transfected the mammalian expression constructs of pCDNA3-Gab1 into HEK-293 T cells and, prior to harvesting the proteins, the cells were treated with pervanadate or retinal *ex vivo* explants treated with pervanadate. The cell lysates were subjected to GST pull-down assay with either GST-PTP1B-WT or GST-PTP1B-D181A fusion proteins followed by immunoblot analysis with anti-Gab1 antibody. We observed that Gab1 specifically bound to PTP1B-D181A mutant, but not by wild type PTP1B (Figure 5A). These results suggest that Gab1 may be a substrate of PTP1B. In addition, we have also examined the association between Gab1 and the substrate-trapping mutant of PTP1B by immunofluorescence on confocal microscopy as an independent confirmation that the association occurred *in vivo* and not after lysis (Figure 5B). Our results indicate a colocalization of Gab1 with mutant PTP1B.

### PTP1B dephosphorylates Gab1 *in vitro*

To determine whether PTP1B dephosphorylates Gab1 *in vitro*, we expressed the Myc-tagged full-length Gab1 in HEK-293 T cells and the proteins were subjected to immunoprecipitation with anti-Myc tag antibody. The immune complexes were incubated in the presence of either wild type PTP1B or catalytically inactive mutant D181A-PTP1B (GST-fusion proteins) for 30 min at 30°C. At the end of incubation, the immunoprecipitates were washed and subjected to immunoblot analysis with anti-PY99 and anti-Myc antibodies. The results indicate that PTP1B completely dephosphorylated Gab1 and the mutant protein failed to dephosphorylate Gab1 (Figure 5C). The Myc tag blot shows the presence of Gab1 in all the immunoprecipitates (Figure 5C). The blot was also reprobed with anti-GST antibody to ensure equal amount of PTP1B fusion protein in all lanes (Figure 5C). This experiment shows that PTP1B can dephosphorylate Gab1 *in vitro*.

### Gab1 phosphorylation is required for PTP1B binding

**Figure 6:**
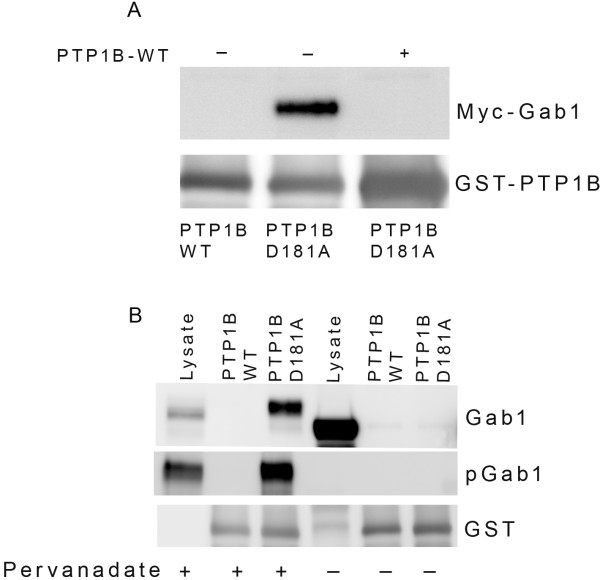
**Gab1 phosphorylation is required for PTP1B binding.** Myc-tagged Gab1 was expressed into HEK-293 T cells followed by pervanadate treatment. The proteins were incubated either in the presence or absence of wild type GST-PTP1B followed by pull-down assay with either wild type PTP1B or mutant PTP1B. The bound proteins were subjected to immuno blot analysis with anti-Myc antibody (**A**). The blot was reprobed with anti-GST antibody to ensure equal amount of fusion in each pull-down (**A**). Myc-tagged Gab1 expressed in HEK-293 T were either treated or untreated with pervanadate. Pervanadate treated or untreated samples were subjected to GST pull-down assay with either wild type PTP1B or mutant PTP1B followed by immuno blot analysis with either Gab1 or pGab1 (**B**). The blot was reprobed with anti-GST antibody to ensure equal amount of fusion in each pull-down (**B**).

To rule out the possibility that Gab1 is non-specifically binding to PTP1B-D181A mutant, but not to wild type PTP1B, we expressed Myc-tagged Gab1 in HEK-293 T cells and prior to harvesting the proteins the cells were treated with pervanadate. The lysates were incubated with or without wild type PTP1B prior to pull-down assays with either wild type PTP1B or PTP1B-D181A mutant. The results indicate that binding of Gab1 to PTP1B-D181A mutant, but not wild type PTP1B (Figure 6A). However, lysates treated with wild-type PTP1B followed by pull down assays with PTP1B-D181A mutant failed to bring down the Gab1 (Figure 6A). These results clearly suggest that the binding of Gab1 to PTP1B mutant is phosphorylation-dependent and it is not due to non-specific interaction.

In the second approach we expressed Myc-tagged Gab1 in HEK-293 T cells and the cells were treated in the presence or absence of pervanadate. The lysates were subjected to GST pull-down assays with either wild type PTP1B or PTP1B-D181A mutant followed by immune blot analysis with anti-Myc and anti-pGab1 antibodies. The results indicate the binding of Gab1 to PTP1B-D181A mutant only from the cells that were treated with pervanadate (Figure 6B). Pull-downs immunoblotted with anti-pGab1 antibody clearly suggest that the binding of Gab1 to PTP1B mutant is phosphorylation-dependent as we failed to recover the Gab1in PTP1B-D181A pull-down in the absence of its phosphorylation (Figure 6B).

### Binding site of PTP1B on Gab1

**Figure 7:**
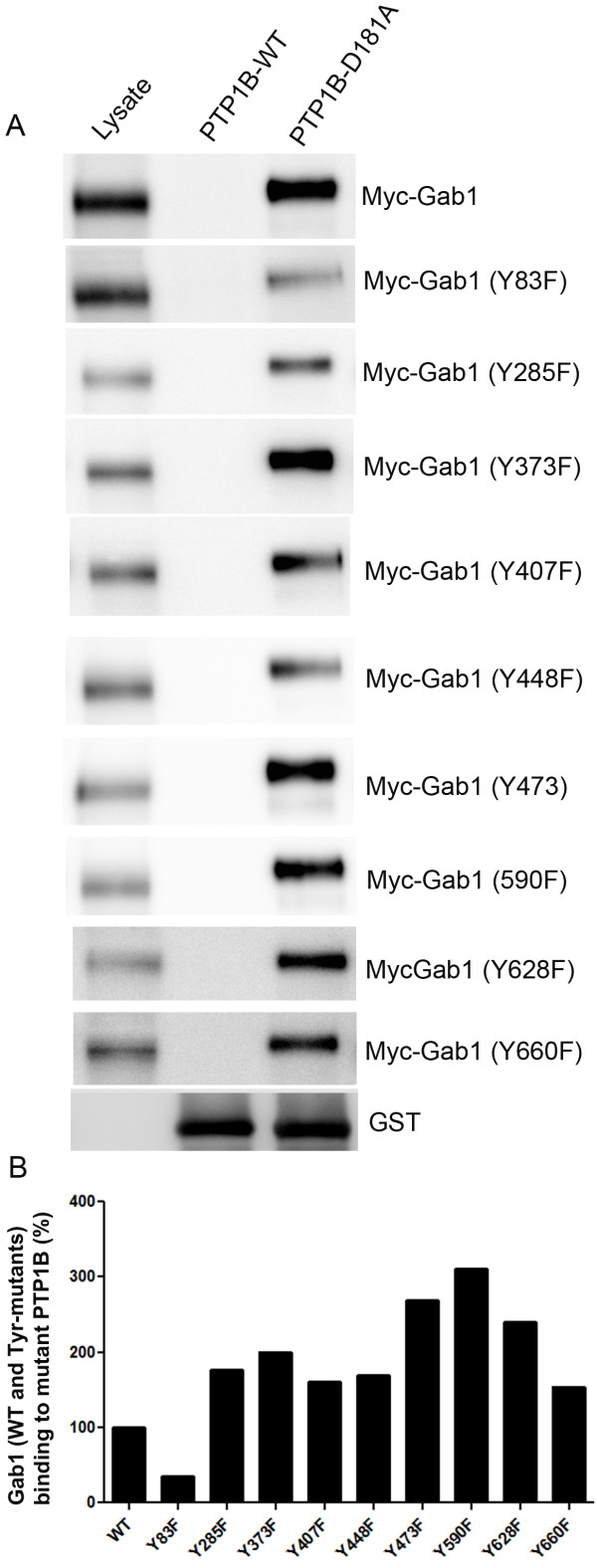
**Interaction of wild type and phosphorylation deficient mutants of Gab1 with substrate trapping mutant of PTP1B.** Myc-tagged wild type and various phosphorylation deficient mutants (Y83F; Y285F; Y373F; Y407F; Y448F; Y473F; Y590F; Y628F; Y660F) were expressed in HEK-293 T cells followed by pervanadate treatment of cells as described in methods. Proteins from wild type and various mutants of Gab1 were incubated with either wild type or mutant PTP1B followed by GST pull-down assay and the bound proteins were subjected to immunoblot analysis with anti-Myc antibody (**A**). The blot was reprobed with anti-GST antibody to ensure equal amount of fusion in each pull-down (A). Denistomeric analysis of immunoblots was performed in the linear range of detection and the binding of various tyrosine mutants of Gab1 to mutant PTP1B was normalized to their respective loading control (lysate) (**B**). The wild type Gab1 binding to mutant PTP1B was set as 100% (**B**).

To determine which phosphorylation site on Gab1 is required for PTP1B binding; we expressed Myc-tagged wild type and various phosphorylation deficient mutant Gab1 constructs into HEK-293 T cells. These mutants include consensus SH2-domain binding sites of Crk/PLCγ (Y83F; Y285F; Y373F and Y407F), p85 (Y448F; Y473F and Y590F) and SHP2 (Y628F and Y660F). The results indicate that none of the mutants could abolish the binding of Gab1 to PTP1B-D181A mutant (Figure 7A). However, when we normalized the binding of various tyrosine mutants of Gab1 to PTP1B with the loading control, the Gab1-Y83F mutant exhibited a reduced binding interaction (35% compared to 100% loading control) with PTP1B. The binding of other mutants with PTP1B were either higher or comparable to wild type control (Figure 7B). These results further suggest that other regions in the Gab1 may also be required in addition to the phosphorylation sites.

**Figure 8:**
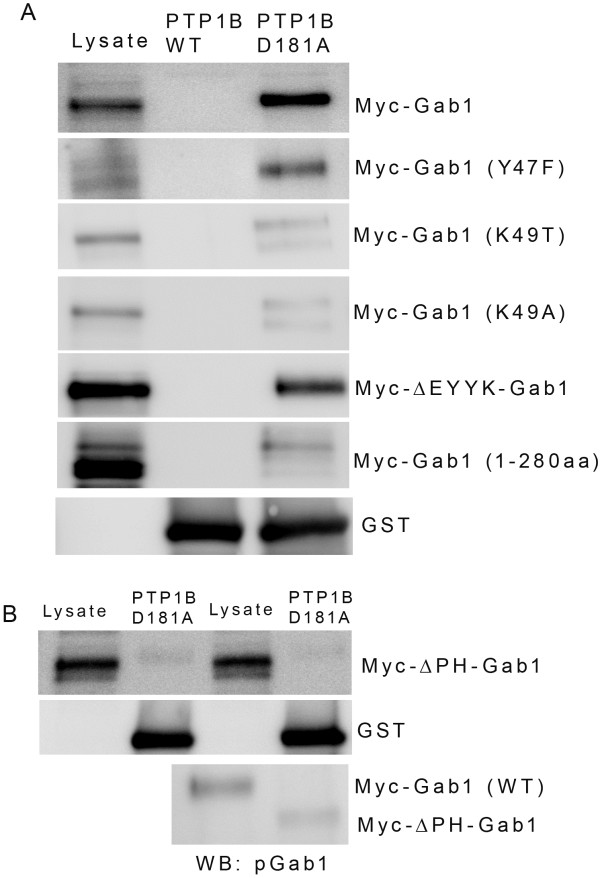
**Interaction of various mutants and deletion constructs of Gab1 with substrate trapping mutant of PTP1B.** Wild type Myc-tagged Gab1, Myc-Gab1 (Y47F), Myc-Gab1 (K49T), Myc-Gab1 (K49A), Myc-Gab1 (ΔEYYK) and truncated version of Myc-Gab1 (1–280 amino acids) constructs were expressed in HEK-293 T cells followed by pervanadate treatment. Pervanadate treated samples were subjected to GST pull-down assay with either wild type PTP1B or mutant PTP1B followed by immunoblot analysis with anti-Myc antibody (A). The blot was reprobed with anti-GST antibody to ensure equal amount of fusion in each pull-down (**A**). PH domain is necessary for the interaction of Gab1 with substrate trapping mutant of PTP1B. Myc-tagged **Δ**PH-Gab1 was expressed in HEK-293 T cells followed by pervanadate treatment. Pervanadate treated samples were subjected to GST pull-down assay with either wild type PTP1B or mutant PTP1B followed by immunoblot analysis with anti-Myc antibody (**B**). The blot was reprobed with anti-GST antibody to ensure equal amount of fusion in each pull-down (B). Proteins from wild type and **Δ**PH-Gab1 was subjected to immunoprecipitation with anti-Myc antibody followed by immunoblot analysis with anti-pGab1 antibody (**B**, bottom panel).

It has been previously shown that PTP1B displayed selectivity for the protein substrates containing the (E/D-pY-pY-(R/K) motif [41]. Examination of the Gab1 sequence clearly indicates that it has a E-Y-Y-K motif between amino acids 46 and 49 (Table 1). The phosphorylation site prediction program [42] indicate that this site is unlikely to be phosphorylated. However, we have created mutations in this site and examined the binding of Gab1 to mutant PTP1B. Substitution of Y47F in Gab1 is still able to bind to PTP1B-D181A mutant (Figure 8A), however, substitution of either Y48F or Y47/48 F Gab1 constructs were failed to express the detectable protein in HEK-293 T cells (data not shown). It has been shown previously that JAK2 (EYYK), but not JAK1 (EYYT) is the substrate of PTP1B, suggesting the importance of lysine in the binding interaction with substrate mutant trap of PTP1B [41]. Therefore, we substituted the lysine 49 with threonine (K49T) or alanine (K49A), and examined the binding of these Gab1-mutants with PTP1B-181A mutant. Our results indicate still a very weak binding of these mutants with PTP1B-D181A mutant (Figure 8A). To determine whether the EYYK motif in Gab1 is an absolute requirement for PTP1B binding, we deleted the first 49 amino acids of Gab1 (ΔEYYK ) and expressed the protein from 50–695 amino acids. The results indicate that **Δ**EYYK-Gab1 binds to PTP1B-D181A mutant similar to wild type Gab1 (Figure 8A). These results suggest that binding site of PTP1B on Gab1 may be other than EYYK and the weak binding observed with K49T/A mutant could be due to competition between PTP1B and EYYK and other unidentified binding site on Gab1.

**Table 1:** Prediction of tyrosine phosphorylation on tyrosine residues in Gab1

**Position of Tyr**	**Sequence**^**1**^	**Score**^**2**^	**Prediction**
24	KLKRYAWKR	0.050	
47	DVLEYYKND	0.279	
48	VLEYYKNDH	0.279	
83	FENSYIFDI	0.687	“Y”
95	DRIFYLVAD	0.040	
162	DPPPYQVIS	0.145	
183	DPQDYLLLI	0.078	
242	QQMMYDCPP	0.070	
259	ESSLYNLPR	0.377	
265	LPRSYSHDV	0.007	
285	DGELYTFNT	0.896	“Y”
307	VSISYDIPP	0.487	
317	PGNTYQIPR	0.498	
373	TDSSYCIPP	0.909	“Y”
407	SQDCYDIPR	0.908	“Y”
428	FHSQYKIKS	0.419	
448	LDENYVPMN	0.932	“Y”
473	QEPNYVPMT	0.980	“Y”
590	SEENYVPMN	0.991	“Y”
628	KQVEYLDLD	0.792	“Y”
660	ERVDYVVVD	0.961	“Y”

^1^The amino acid sequence surrounding the Tyr (Y).

^2^Phosphorylation scores were calculated based on the phosphorylation site prediction program [42]. Scores above 0.5 are deemed to be possible phosphorylation sites and the higher the score, the more likely a particular site will be phosphorylated. *Mus musculus* Gab1 protein sequence was used for the analysis [accession number AJ 250669].

To identify the binding site on Gab1, we expressed the Gab1 protein in HEK-293 T cells from 1–280 amino acids which contain only one likely phosphorylated tyrosine residue 83. This truncated protein is able to interact with PTP1B-D181A mutant (Figure 8A). Our results on Y83F mutant did not abolish the binding interaction between Gab1 and PTP1B-D181A mutant; it is likely that the binding is dictated by the cooperative tyrosine phosphorylation and a region between 50–280 amino acids in Gab1. Examinations of region between 50–280 amino acids clearly indicate the presence of PH domain (1–116 amino acids). When we deleted the PH domain from the Gab1, we failed to observe the interaction with the PTP1B-D181A mutant (Figure 8B), even though the deleted PH domain of Gab1 is tyrosine phosphorylated (Figure 8B, bottom panel). These results clearly suggest that the tyrosine phosphorylation and PH domain of Gab1 is required for substrate recognition of PTP1B.

### Increased Gab1 phosphorylation in PTP1B knockout mouse retinas and a PTP1B inhibitor-treated retinas

**Figure 9:**
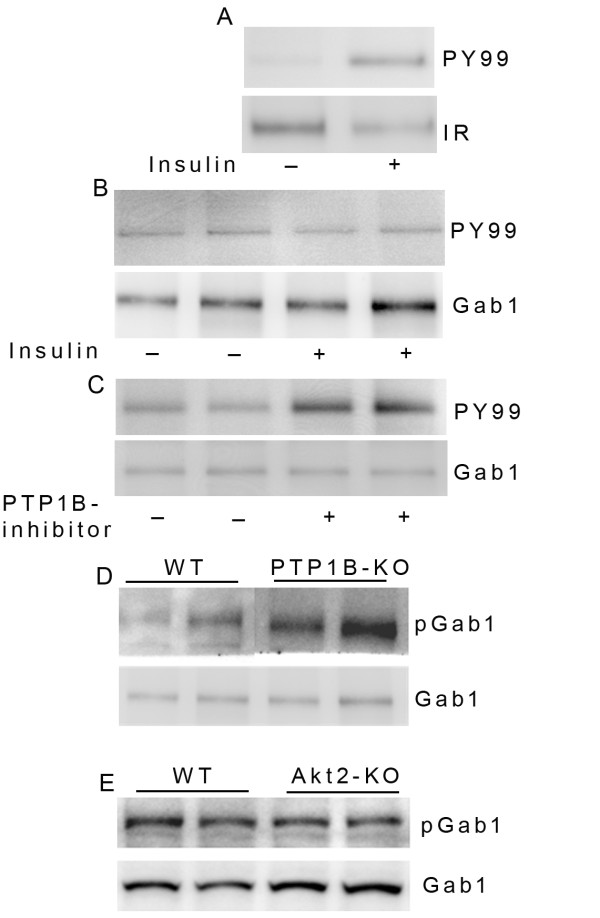
**Enhanced Gab1 tyrosine phosphorylation in PTP1B inhibitor-treated and PTP1B knockout mouse retinas.** Retinal *ex vivo* explants from dark adapted rats were incubated with or without 1 μM insulin (5 min at RT) or DMSO (30 min at RT) or 100 μM PTP1B inhibitor (30 min at RT). Anti-IRβ and anti-Gab1 immunoprecipitates were immunoblotted with PY99 (**A, B, C**) and reprobed the blots with IRβ (**A**) or Gab1 (**B**, **C**) antibodies. Wild type, PTP1B and Akt2 knockout mouse retinal proteins were subjected to immunoblot analysis with anti-pGab1 and Gab1 (**D, E**) antibodies. The panel D, upper portion was stitched together from two areas of the blot representing the same migration window. Two independent mouse retinas were used in experiments described in Panel B, C, D and E.

Insulin-induced Gab1 tyrosine phosphorylation and association of Gab1 with Src homology-2 (SH2) domain-containing proteins has been reported [43]. In this study we examined the effect of insulin on Gab1 tyrosine phosphorylation by incubating retinal *ex vivo* explants from dark-adapted rats with insulin for 5 min. Retinal proteins were subjected to immunoprecipitation with either anti-Gab1 or anti-IRβ antibodies followed by immunoblot analysis with PY99 antibody. The blot was reprobed with anti-Gab1 and anti-IRβ antibodies. The results indicate that insulin failed to induce the tyrosine phosphorylation of Gab1 in retinal *ex vivo* explants (Figure 9B). Insulin-induced tyrosine phosphorylation of insulin receptor confirms that the insulin used in the retinal *ex vivo* system is functional (Figure 9A). To determine the effect of PTP1B inhibition on tyrosine phosphorylation of Gab1, we incubated the retinal *ex vivo* explants from dark-adapted rats with PTP1B specific inhibitor for 30 min. Retinal proteins were subjected to immunoprecipitation with anti-Gab1 followed by immunoblot analysis with PY99 antibody. The blot was reprobed with anti-Gab1 antibody. The results indicate that inhibition of PTP1B resulted in increased tyrosine phosphorylation of Gab1 and the total levels of Gab1 remains same in both inhibitor treated and un-treated (DMSO) conditions (Figure 9C). This experiment suggests that PTP1B regulates the phosphorylation state of Gab1. In a separate approach, wild type and PTP1B knockout mouse retinal proteins were subjected to immunoblot analysis with anti-pGab1-Tyr^627^ and anti-Gab1 antibodies. The results indicate an increased level of Gab1 phosphorylation in PTP1B knockout mouse retinas compared to wild type retinas (Figure 9D). The effect of PTP1B on Gab1 phosphorylation is specific as immunoblots carried out with Akt2 knockout mouse retinas did not show any increase in Gab1 phosphorylation from its wild type littermates (Figure 9E). These results suggest that PTP1B regulates the Gab1 phosphorylation *in vivo*.

## Discussion

Tyrosine kinase receptors and downstream pathways used in growth factor signaling are shared by oxygen free radical signaling [44]. Different growth factor receptors and cytokines are known to activate the tyrosine phosphorylation of Gab1 which in turn activates different signaling pathways, including PI3K/Akt [3,9,45,46], ERK [13,33] and JNK [10,47]. In this study we observed that H_2_O_2_ stimulates the tyrosine phosphorylation of retinal Gab1. On the other hand, light stress decreased the binding of PI3K to Gab1 (data not shown) suggesting a loss of Gab1 phosphorylation under light stress. It has been shown previously that H_2_O_2_ stimulates the tyrosine phosphorylation of Gab1 in wild type mouse embryonic fibroblasts and the activated Gab1 recruits molecules such as SHP2, PI3K, and Shc [38]. These studies clearly indicate that Gab1 is a component of oxidative stress signaling [38]. Gab1 is also associated with similar proteins following stimulation with EGF, insulin, NGF, or HGF [3,8-11]. The Gab1/PI3K interaction with subsequent activation of Akt activation has been shown to protect the PC12 cells or sympathetic neurons from apoptosis induced by serum deprivation [9,46].

The phosphorylation status of Gab1 after H_2_O_2_ treatment has been previously explained due to the activation of EGFR [38]. It is interesting to note in this study that Gab1 is expressed in rod inner segments and its state of phosphorylation is light-dependent. In retina, EGFR expression has shown to be during the first two postnatal weeks in Müller glia and declines as the retina matures; in response to light-damage, EGFR expression is upregulated which has shown to be close to neonatal retina [48]. Insulin-induced Gab1 tyrosine phosphorylation and association of Gab1 with Src homology-2 (SH2) domain-containing proteins has been reported [43]. Retinal *ex vivo* explants treated with insulin did not induce the tyrosine phosphorylation of Gab1. These studies suggest that light-induced tyrosine phosphorylation of Gab is regulated through an unknown mechanism not known at this time.

It has also been suggested that there is also inactivation of phosphatases in oxidative signaling [38]. Hydrogen peroxide can irreversibly inactivate PTP1B *in vivo* and contribute to EGFR phosphorylation after EGF treatment [49]. Several studies in literature indicate that PTP1B is somewhat promiscuous in its substrate preference *in vitro*, dephosphorylating a wide variety of protein and peptide substrates with widely varying *K*_m_ values [50-52]. Substrate-trapping mutants of PTPs have been shown to be ideal reagents for substrate identification. It was demonstrated that such mutants of PTPs can be produced by mutation of Asp to Ala in the conserved WPD loop [40]. The Asp to Ala mutants of PTP1B, TC-PTP, PTPH1, and PTP-PEST helped identify EGFR, p52^shc^, VCP (p97/CDC48), TYK2 and JAK2, and p130^Cas^ as candidate substrates, respectively [40,41,53-55]. We found that Gab1 stably associates with mutant PTP1B in a tyrosine phosphorylation-dependent manner. These observations suggest that Gab1 could be a putative substrate of PTP1B. Consistent with this observation, Gab1 has previously been identified as one of the PTP1B substrates by Bayesian Integration of Proteome [56].

Mutational analysis of various tyrosine residues in Gab1 indicated that none of the mutants abolished the binding interaction with PTP1B. However, we found a decreased binding of Y83F with PTP1B. This result is of particular interest since one of the only two Gab1 mutations associated with cancer is Y83C [57-59]. Further studies are required to understand the interaction between PTP1B and Gab1-Y83 in tumorigenesis. Our studies also suggest that a region from 1–280 amino acids in Gab1 is required for PTP1B binding.

It is interesting to note that there are no studies available on the role of Gab1 in the retina, however, deletion of Gab1 binding protein Shp2 (src-homology phosphotyrosyl phosphatase 2) has been shown result in retinal degeneration [60]. Experiments described in this manuscript suggest that PTP1B negatively regulates the Gab1 phosphorylation. Clear evidence comes from the light/dark experiments where higher phosphorylation of Gab1 in light-adapted conditions was correlated with significantly decreased levels of PTP1B and in dark-adapted conditions, higher PTP1B levels correlated with decreased levels of Gab1 phosphorylation. Such a negative relationship has been observed previously between PTP1B and Gab1 in which PTP1B-mediated dephosphorylation of Gab1 has been shown to affect its EGF-induced association with the phosphatase SHP2 [56]. Increased Gab1 phosphorylation in PTP1B inhibitor-treated retinas and PTP1B knockout mouse retinas further strengthen the evidence that PTP1B regulates the phosphorylation state of Gab1 *in vivo.* Our laboratory has previously reported that retinas deficient of PTP1B are resistant to light damage compared to wild type mice [61]. We have also reported that intravenous injection of an allosteric inhibitor of PTP1B protects rats against light stress-induced retinal degeneration through the protection of IR phosphorylation [61]. We have also reported enhanced insulin receptor neuroprective signaling in PTP1B deficient mice [61]. Since Gab1 is negatively regulated by PTP1B, a part of the retinal neuroprotective effect we have observed previously in PTP1B deficient mice could be contributed by Gab1 as well. Further studies are required to determine the Gab1-medited neuroprotective survival signaling in the retina.

## Conclusions

In this study we have identified a physical and functional interaction between Gab1 and PTP1B. We also found that Gab1 undergoes a light-dependent phosphorylation and PTP1B regulates the phosphorylation state of Gab1. Consistent with these observations, we found an enhanced Gab1 tyrosine phosphorylation in PTP1B deficient mice and PTP1B-inhibitor treated retinas. Collectively, our data suggest that Gab1 is an endogenous physiological protein substrate of PTP1B.

## Methods

### Materials

Anti-PTP1B antibody was obtained from Epitomics (Burlingame, CA). Polyclonal anti-PTP1B, anti-Gab1 antibodies and phosphatase assay reagents were obtained from Upstate Biotechnology (Lake Placid, NY). Monoclonal PY-99 and polyclonal IR antibody was obtained from Santa Cruz Biotechnology (Santa Cruz, CA). An anti-pGab1 antibody was obtained from Cell Signaling (Beverly, MA). The actin antibody was obtained from Affinity BioReagents (Golden, CO). Quick change site-directed mutagenesis kit was obtained from Stratagene (La Jolla, CA). All other reagents were of analytical grade and from Sigma. The PTP1B inhibitor (3-(3,5-Dibromo-4-hydroxy-benzoyl)-2-ethyl-benzofuran-6-sulfonicacid-(4-(thiazol-2- ylsulfamyl)-phenyl)-amide) was obtained from Calbiochem (San Diego, CA).

### Animals

All animal work was done in strict accordance with the NIH Guide for the Care and Use of Laboratory Animals and the Association for Research in Vision and Ophthalmology on the Use of Animals in Vision Research. All protocols were approved by the IACUC at the University of Oklahoma Health Sciences Center and the Dean McGee Eye Institute. In all experiments, rats and mice were killed by asphyxiation with carbon dioxide before the retinas were harvested. A breeding colony of albino Sprague–Dawley (SD) rats is maintained in our vivarium in cyclic light (5 lux; 12 h on/12 h off). Experiments were carried out on both male and female rats (150–200 g). Breeding colonies of PTP1B and Akt2 knockout mice are maintained in our vivarium. The source of global PTP1B [62] and Akt2 [37] knockout mice have been reported earlier.

### Plasmid construction and transfection

The mammalian expression construct of Gab1 was kindly provided by Dr. Ute Schaeper (Berlin, Germany). The Myc-tagged form of full-length Gab1 was generated by adding the Myc-epitope at its C-terminus by PCR and the cDNA encoding Myc-tagged Gab1 was cloned into pCDNA3 vector. All constructs that involved PCR were verified by DNA sequencing. Human embryonic kidney cells (HEK-293 T) were grown in 10% FBS and transfected with 10 μg of DNA in 10-cm plates by calcium phosphate method [63]. Retinal PTP1B was obtained by PCR of reverse transcribed mouse retinal RNA using a 5′ and 3′ oligonucleotide designed based on mouse PTP1B cDNA sequence [64] (accession number NP_035331) (sense: GAA TTC ATG GAG ATG GAG AAG GAG TTC GAG; antisense: GTC GAC TCA GTG AAA ACA CAC CCG GTA GC). Site-directed mutagenesis was carried out according to the method described earlier [65]. Gab1-Y83F; sense: TTT GAA AAC AGC TTT ATC TTT GAT ATC AAC; antisense: GTT GAT ATC AAA GAT AAA GCT GTT TTC AAA; Gab1-Y285F; sense GAC GGG GAG CTG TTC ACC TTT AAC ACC CCA; antisense: TGG GGT GTT AAA GGT GAA CAG CTC CCC GTC; Gab1-Y373F; sense ACT GAC AGC AGT TTC TGT ATC CCT CCT CCA; antisense: TGG AGG AGG GAT ACA GAA ACT GCT GTC AGT; Gab1-Y407F; sense TCT CAA GAT TGC TTT GAT ATT CCA CGG ACC; antisense: GGT CCG TGG AAT ATC AAA GCA ATC TTG AGA; Gab1-Y448F; sense: CTG GAT GAG AAC TTC GTT CCC ATG AAC CCC; antisense: GGG GTT CAT GGG AAC GAA GTT CTC ATC CAG; Gab1-Y473F, sense: CAG GAG CCA AAC TTT GTG CCA AATG ACC CCA; antisense: TGG GGT CAT TGG CAC AAA GTT TGG CTC CTG; Gab1-Y590F; sense: AGT GAA GAG AAC TTT GTC CCC ATG AAT CCA; antisense: TGG ATT CAT GGG GAC AAA GTT CTC TTC ACT; Gab1-Y628F; sense AAA CAA GTC GAA TTC CTG GAT TTA GAC; antisense: GTC TAA ATC CAG GAA TTC GAC TTG TTT; Gab1-Y660F; GAG AGG GTG GAT TTC GTT GTG GTG GAC CAA; antisense: TTG GTC CAC CAC AAC GAA ATC CAC CCT CTC; Gab1-R49T; sense GTC CTG GAG TAT TAC ACA AAC GAT CAT GCC GCA; antisense: GGC ATG ATC GTT TGT GTA ATA CTC CAG GAC; Gab1-R49A; sense: GTC CTG GAG TAT TAC GCA AAC GAT CAT GCC; antisense: GGC ATG ATC GTT TGC GTA ATA CTC CAG GAC; Gab1-Y47F: sense GAT GTC CTG GAG TTT TAC AAA AAC GAT CAT; antisense: ATG ATC GTT TTT GTA AAA CTC CAG GAC ATC. The PTP1B binding motif on Gab1 (ΔEYYK) was deleted and the expression construct (49–695 amino acids) was generated using the following primers: sense: GAA TTC ACC ATG GAC ATC TGT GGA TTC AAT CCC ACA G GAA TTC ACC ATG AAC GAT CAT GCC AAG AAG CC and antisense: GGA TCC CTT CAC ATT CTT GGT GGG TGT CTC GG. Truncated versions of Gab1 were also generated using the following primers: Gab1 (1–280 amino acids) sense, GAA TTC C ACC ATG AGC GGC GGC GAA GTG GTT TGC TCG GG and antisense: GGA TCC GGC CTC CGT GCT TGA TGG GGA TTC C. The PCR products were cloned into TOPO sequencing vector (Invitrogen) and the sequences were verified by DNA sequencing. The inserts were excised as EcoRI/BamHI and cloned into C-terminal Myc-tagged pCDNA3 vector. The primers used in the site-directed mutagenesis are as follows: PTP1B-D181A (sense: ACC ACA TGG CCT GCC TTT GGA GTC CCC; antisense: GGG GAC TCC AAA GGC AGG CCA TGT GGT). The PCR products were cloned into TOPO sequencing vector (Invitrogen) and the sequences were verified by DNA sequencing. The WT and mutant cDNA were excised from the sequencing vector as EcoRI/SalI and cloned into GST fusion vector, pGEX-4 T1. Site-directed mutagenesis was carried out according to the method described earlier [65]. The cloning and expression of N-SH2 domain of p85 subunit of PI3K has been reported previously [66].

### Expression of GST-fusion proteins

An overnight culture of *E.coli* BL21 (DE3) (pGEX-PTP1B and pGEX-PTP1B-D181A) was diluted 1:10 with 100 μg/ml ampicillin, grown for 1 hr at 37°C, and induced for another hour by addition of IPTG to 1 mM. Bacteria were sonicated three times for 20 s each time in lysis buffer containing 10 mM imidazole-HCl (pH7.2), 1 mM EDTA, 100 mM NaCl, 1 mM dithiothreitol, and 1% Triton X-100. Lysates were clarified by centrifugation, and the supernatants were incubated with 500 μl of 50% glutathione-coupled beads (Amersham Pharmacia) for 30 min at 4°C. The GST-PTP1B fusion proteins were washed in lysis buffer and eluted twice with 1 ml of 5 mM reduced glutathione (Sigma) in phosphatase buffer [20 mM Tris (pH 7.4), 5% glycerol, 0.05% Triton X-100, 2.5 mM MgCl2, aprotinin (2 μg/ml) and leupeptin (5 μg/ml)]. Glycerol was added to a final concentration of 33% (vol/vol), and aliquots of enzyme were stored at −20°C.

### Substrate trapping *in vitro*

The GST fusion proteins were expressed in *E.coli* and purified on glutathione-Sepharose beads according to the manufacturer’s instructions. Pervanadate stock solution (1 mM) was prepared [67] by adding 10 μl of 100 mM vanadate and 50 μl of 100 mM hydrogen peroxide (diluted from 30% stock in 20 mM HEPES, pH 7.3) to 940 μl of H_2_O. Excess hydrogen peroxide was removed by adding catalase (100 μg/ml; final concentration = 260 units/ml) 5 min after mixing the vanadate and hydrogen peroxide. The pervanadate solutions were used within 5 min to minimize decomposition of the vanadate-hydrogen peroxide complex. Retinal *ex vivo* explants or mammalian cells were treated with 1 mM pervanadate for 30 min, washed with phosphate-buffered saline, and lysed in substrate-trapping buffer [40]. The lysates were incubated for 2 h at 4°C with either GST or GST-PTP1B-WT or GST-PTP1B-D181A mutant fusion proteins bound on beads, then the beads were washed 4 times with trapping buffer. Bound proteins were resolved by SDS-PAGE and blotted onto nitrocellulose membranes. Blots were then incubated with anti-PY99 or anti-Gab1 antibodies and developed by ECL.

### PTP1B Activity assay

The *in vitro* PTP activity assay was conducted based on a previously published protocol using the peptide RRLIEDAE_P_YAARG (Upstate Biotechnology) [68]. The reaction was carried out in a 60 μL volume of PTP assay buffer [100 mm HEPES (pH 7.6), 2 mm EDTA, 1 mm dithiothreitol, 150 mm NaCl, 0.5 mg/ml bovine serum albumin] at 30°C. At the end of the reaction, 40 μL aliquots were placed in a 96-well plate, 100 μL of Malachite Green Phosphatase reagent (Upstate Biotechnology) were added, and absorbance was measured at 630 nm.

### Retinal *Ex-vivo* organ cultures

Retinal *ex vivo* organ cultures were carried out as previously described [65]. Retinas were removed from Sprague–Dawley albino rats that were born and raised in dim cyclic light (5 lux; 12 h ON: 12 h OFF) and incubated for 5 min at 37°C in Dulbecco’s modified Eagle’s (DMEM) medium (Gibco BRL) in the presence or absence of 600 μM H_2_O_2_ or 100 μM PTP1B inhibitor (3-(3,5-Dibromo-4-hydroxy-benzoyl)-2-ethyl-benzofuran-6-sulfonicacid-(4-(thiazol-2- ylsulfamyl)-phenyl)-amide) [61] or DMSO. At the indicated times, retinas were snap-frozen in liquid nitrogen and stored at −80°C until analyzed or lysed in lysis buffer [1% NP 40, 20 mM HEPES (pH 7.4), 2 mM EDTA, phosphatase inhibitors (100 mM NaF, 10 mM Na4P2O7, 1 mM NaVO3, and 1 mM molybdate), and protease inhibitors (10 μM leupeptin, 10 μg/ml aprotinin and 1 mM PMSF)].

### Preparation of Rod outer segments

ROS were prepared from rat retinas using a discontinuous sucrose gradient as previously described [66]. Retinas were homogenized in 4.0 ml of ice-cold 47% sucrose solution containing 100 mM NaCl, 1 mM EDTA, 1 mM PMSF, and 10 mM Tris–HCl (pH 7.4). Retinal homogenates were transferred to 15-ml centrifuge tubes and sequentially overlaid with 3.0 ml of 42%, 3.0 ml of 37%, and 4.0 ml of 32% sucrose dissolved in buffer A [10 mM Tris–HCl (pH 7.4) containing 100 mM NaCl and 1 mM EDTA]. The gradients were spun at 82,000 × g for 1 h at 4°C. The 32/37% interfacial sucrose band containing ROS membranes was harvested and diluted with buffer A, and centrifuged at 27,000 × g for 30 min. The ROS pellets were resuspended in buffer A, and stored at −20°C. All protein concentrations were determined by the BCA reagent following the manufacturer’s instructions.

## Abbreviations

Gab1: Grb2-associated binding protein 1; PTP1B: Protein tyrosine phosphatase-1B; IR: Insulin receptor; PI3K: Phosphoinositide 3-kinase; Shp2: Src-homology phosphotyrosyl phosphatase 2; SDM: Site-directed mutagenesis

## Competing interests

The authors declare that they have not competing interests.

## Authors’ contribution

RR contributed to the design of the study. AR, AD and RR performed the experiments and analysis of data. RR contributed to the writing of the manuscript. All authors read and approved the final version of this manuscript.
